# Tuberculosis, Human Immunodeficiency Virus, and the Association With Transient Hyperglycemia in Periurban South Africa

**DOI:** 10.1093/cid/ciz928

**Published:** 2019-09-26

**Authors:** Mmamapudi Kubjane, Natacha Berkowitz, Rene Goliath, Naomi S Levitt, Robert J Wilkinson, Tolu Oni

**Affiliations:** 1 Division of Public Health Medicine, School of Public Health & Family Medicine, University of Cape Town, Cape Town, South Africa; 2 Wellcome Centre for Infectious Disease Research in Africa, Institute of Infectious Disease and Molecular Medicine, and Department of Medicine, University of Cape Town, Observatory, South Africa; 3 Division of Diabetes and Endocrinology, Department of Medicine, Groote Schuur Hospital, Cape Town, South Africa and Chronic Disease Initiative for Africa; 4 The Francis Crick Institute, London, United Kingdom; 5 Department of Infectious Diseases, Imperial College London, United Kingdom; 6 Medical Research Council Epidemiology Unit, University of Cambridge, Cambridge, United Kingdom

**Keywords:** tuberculosis, HIV, diabetes, infectious disease, NCD, multimorbidity

## Abstract

**Background:**

Diabetes mellitus (DM) increases tuberculosis (TB) risk. We assessed the prevalence of hyperglycemia (DM and impaired glucose regulation [IGR]) in persons with TB and the association between hyperglycemia and TB at enrollment and 3 months after TB treatment in the context of human immunodeficiency virus (HIV) infection.

**Methods:**

Adults presenting at a Cape Town TB clinic were enrolled. TB cases were defined by South African guidelines, while non-TB participants were those who presented with respiratory symptoms, negative TB tests, and resolution of symptoms 3 months later without TB treatment. HIV status was ascertained through medical records or HIV testing. All participants were screened for DM using glycated hemoglobin and fasting plasma glucose at TB treatment and after 3 months. The association between TB and DM was assessed.

**Results:**

Overall DM prevalence was 11.9% (95% confidence interval [CI], 9.1%–15.4%) at enrollment and 9.3% (95% CI, 6.4%–13%) at follow-up; IGR prevalence was 46.9% (95% CI, 42.2%–51.8%) and 21.5% (95% CI, 16.9%–26.3%) at enrollment and follow-up. TB/DM association was significant at enrollment (odds ratio [OR], 2.41 [95% CI, 1.3–4.3]) and follow-up (OR, 3.3 [95% CI, 1.5–7.3]), whereas TB/IGR association was only positive at enrollment (OR, 2.3 [95% CI, 1.6–3.3]). The TB/DM association was significant at enrollment in both new and preexisting DM, but only persisted at follow-up in preexisting DM in patients with HIV-1 infection.

**Conclusions:**

Our study demonstrated high prevalence of transient hyperglycemia and a significant TB/DM and TB/IGR association at enrollment in newly diagnosed DM, but persistent hyperglycemia and TB/DM association in patients with HIV-1 infection and preexisting DM, despite TB therapy.

## Teaser

This study found a significant association between tuberculosis (TB) and diabetes mellitus (DM)/impaired glucose regulation at baseline, transient in newly diagnosed DM. Hyperglycemia was persistent even after 3 months of TB therapy in preexisting (treated) DM, with the TB/DM association remaining significant overall, and stronger in participants with HIV-1 infection.

Mortality in South Africa is characterized by concurrent infectious and noncommunicable diseases. The 2015 mortality report confirms this transition, with tuberculosis (TB) and diabetes mellitus (DM) ranked the first and second leading causes of death, while human immunodeficiency virus type 1 (HIV-1) was ranked fifth [1]. Although the role that HIV-1 plays as a significant driver of the TB epidemic is well recognized, the emerging and rapidly growing burden of DM, another TB risk factor, presents another challenge to TB control.

An increasing body of research shows an association between DM and TB [2, 3]. This association is becoming more apparent due to the epidemiological transition as DM is growing rapidly in settings where HIV-1 and TB epidemics persist. Diabetes increases the risk of developing TB and is also associated with adverse treatment outcomes, including death [3–6].

Tuberculosis increases insulin resistance and stress-induced hyperglycemia that may revert to normal during treatment [7, 8]. Therefore, testing for DM in persons with recently diagnosed TB may lead to misclassification of transient hyperglycemia as DM, and overestimation of the diabetes/TB association. Testing for DM in TB patients is recommended, with confirmatory tests after 2–3 months of TB treatment initiation [9, 10]; however, the optimal time for screening and implications for clinical management are unknown.

The interplay between TB and DM in the context of HIV-1 infection remains unclear due to limited data particularly in the African context. A recent review that only included 3 African studies reported odd ratios (ORs) of TB in DM patients ranging from 0.9 (95% confidence interval [CI], .2–4.6) to 10.7 (95% CI, 4.5–26.0) [11]. A previous study in Cape Town demonstrated significant TB and DM association in the group with HIV infection (OR, 2.4 [95% CI, 1.1–5.2]) vs HIV-uninfected patients (OR, 2.4 [95% CI, .9–6.7]) [12]. A study in Tanzania observed a stronger TB/DM association among people living with HIV [13], while another Tanzanian study observed stronger TB/DM association in HIV-1–uninfected persons (OR, 4.2 [95% CI, 1.5–11.6]) compared with people living with HIV (OR, 0.1 [95% CI, .01–1.8]) [14]. These 3 diseases interact, with the potential to influence risk of disease and prognosis and to complicate clinical management [15].

The objective of this study was to assess the association between hyperglycemia and TB, at TB diagnosis, and after 3 months of TB treatment.

## METHODS

### Study Setting and Population

This study was conducted at the largest public-sector TB clinic in Khayelitsha, a periurban township of approximately 390  000 predominantly black Africans, in Cape Town, Western Cape province, where DM, HIV-1, and TB rank as the first, third, and fifth leading causes of death, respectively [1]. The 2012 HIV-1 antenatal prevalence in Khayelitsha was 34% (95% CI, 31.0%–36.6%) (Western Cape Department of Health, Cape Town, South Africa; 2012 Antenatal Survey, unpublished data) and the 2015 TB case notification rate was 917 per 100  000 population (V. de Azevedo, City Health Manager, Cape Town; personal communication), with a 70% HIV-1/TB coinfection rate [16]. The prevalence of diabetes is 13.1% (95% CI, 11.0%–15.1%) [17].

### Study Design and Sampling

A 3-month cohort study on consecutive patients with respiratory symptoms presenting to the clinic from July 2013 to August 2015. Patients were eligible if they provided consent, were ≥18 years of age, and had received <48 hours of TB chemotherapy. Those critically ill and in need of emergency clinical care were ineligible due to inability to provide informed consent. Based on the 4.5% and 1.2% prevalence of diabetes in TB patients and non-TB patients, respectively [13], assuming 80% power and 5% significance level, the required sample size was 798 (n = 399 per group) [18].

### Study Procedures

#### Case Definitions

All participants were tested for TB according to South African guidelines [19]. Samples were analyzed in a centralized national health laboratory. TB cases had a positive GeneXpert result. Non-TB participants were those with a negative GeneXpert result, who after examination by a physician had resolution of respiratory symptoms without TB treatment after 3 months. HIV status and antiretroviral therapy (ART) were ascertained from participants' medical records. For participants with unknown HIV status, voluntary HIV testing was offered, and if found to have HIV infection, they were provided counseling and ART initiation. All participants were tested for DM using fasting plasma glucose (FPG) and glycated hemoglobin (HbA1c). DM was defined as self-reported DM, FPG ≥7.0 mmol/L, or HbA1c ≥6.5%. Impaired glucose regulation (IGR) was defined as FPG 5.5 to <7.0 mmol/L or HbA1c 5.7% to <6.5% [20].

#### Measurements

After TB diagnosis, sputum microscopy was repeated at months 3 and 5 in patients with pulmonary smear-positive TB according to South African guidelines [19]. For DM diagnosis, venous blood was drawn after an overnight fast for FPG. At 3-month follow-up, both DM tests were repeated in all participants. All blood samples were processed on the day of collection at a centralized national health laboratory using standardized operating procedures of the Roche/Hitachi Cobas C311 system analyzer assay [21]. Weight, height, and waist circumference were measured [22]. The body mass index (BMI [kg/m^2^]) was categorized as follows: underweight, <18.5; normal, 18.5–24.9; overweight, 25–29.9; obese, ≥30) [22]. The cutoff point for high waist circumference was ≥94 cm (males) and ≥88 cm (females) [22]. Hypertension was defined as a single measured blood pressure (BP) of systolic BP >140 mm Hg or diastolic BP >90 mm Hg [20], or a preexisting diagnosis.

#### Questionnaire

Socioeconomic, demographic, and chronic medical and medication history for HIV-1, DM, and hypertension were collected using a researcher-administered questionnaire.

### Statistical Analysis

Medians and interquartile ranges (IQRs) and proportions summarized continuous and categorical variables. The χ ^2^ and Fisher exact tests assessed associations between categorical variables, respectively. The Mann-Whitney test was used to compare medians between 2 groups and the Kruskal-Wallis test for >2 groups. A multivariable logistic regression model for TB/DM association was manually built, using forward selection, controlling for potential confounding variables. To retain statistical power in the regression analysis, multiple imputation was used to impute HIV-1 serostatus for 50 participants with unknown HIV-1 status. We conducted sensitivity analysis comparing complete case and imputed analyses for multivariate analysis results on the association between TB and IGR/DM (Supplementary Table 1). Statistical significance was set at *P* < .05. All data were analyzed using Stata version 13.0 software (StataCorp, College Station, Texas).

### Ethical Considerations

This study was approved by the University of Cape Town Human Research Ethics Committee (HREC REF: 403/2011).

## RESULTS

### Study Sample at Enrollment

**Figure 1.**
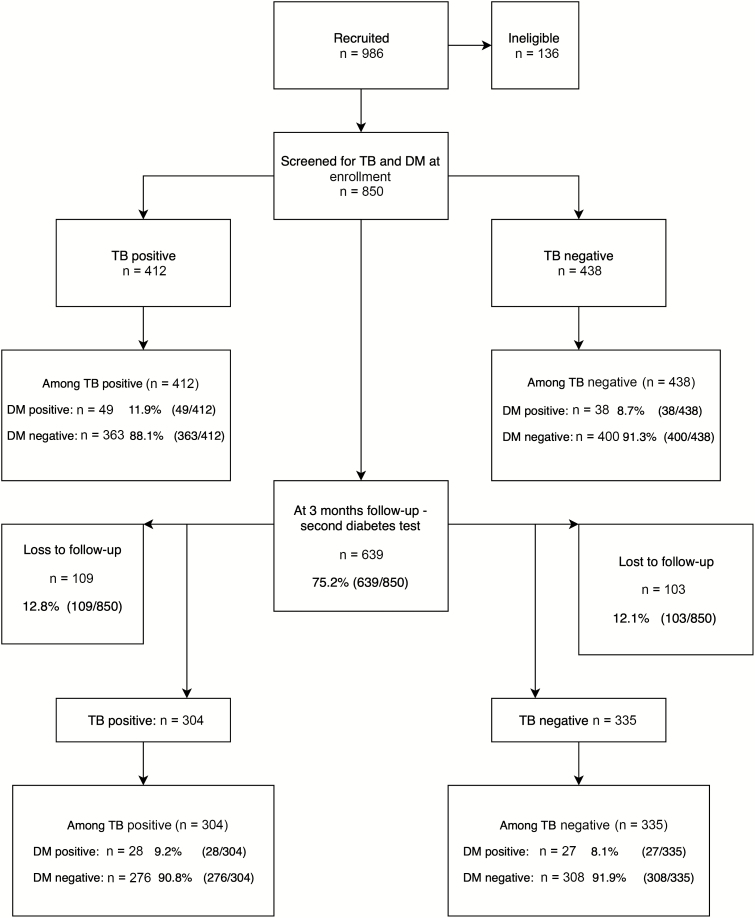
Participant recruitment flow diagram. Abbreviations: DM, diabetes mellitus; TB, tuberculosis.

Nine hundred eighty-six participants were recruited, and 48 participants (4.9%) were excluded as TB could not be confirmed or excluded. A further 88 participants did not complete diabetes screening at enrollment. For the analysis, 850 participants were included: 412 TB cases and 438 non-TB participants (Figure 1).

### Participant Characteristics at Enrollment

Patient characteristics at enrollment have been previously described [12]. The overall median age of participants was 38 (IQR, 31–47) years, with 53% male. Compared to those without TB, TB cases were younger, with a lower prevalence of central obesity, a greater proportion of men, and a higher proportion of previous incarceration (Table 1). Of the 412 TB cases, 9 had rifampicin resistance (2.2%) and did not have diabetes. The overall prevalence of HIV-1 infection was 61.1% (95% CI, 57.8%–64.4%); 67.2% (95% CI, 62.7%–71.8%) among TB cases; and 55.3% (95% CI, 50.1%–59.7%) among non-TB cases [12].

**Table 1.** Characteristics of Participants With and Without Tuberculosis at Enrollment

Characteristic	Without TB (n = 438)	With TB (n = 412)	Total (N = 850)	*P* Value
Age, y				
18–24	16 (3.7)	43 (10.4)	59 (6.9)	**<.001**
25–34	126 (28.8)	151 (36.7)	277 (32.6)	
35–44	126 (28.8)	136 (33.0)	262 (30.8)	
45–54	95 (21.7)	53 (12.9)	148 (17.4)	
≥55	75 (17.1)	29 (7.0)	104 (12.2)	
			850	
Age, y, median (IQR)	41 (32–50)	36 (30–43)	38 (31–47)	**<.001**
			850	
Sex				
Female	224 (51.3)	175 (42.6)	399 (47.0)	**.011** ^a^
			848	
Education level				
Up to primary	130 (30.4)	129 (32.3)	259 (31.3)	.268
Up to secondary	289 (67.7)	257 (64.3)	456 (66.0)	
Higher education	8 (1.9)	14 (3.5)	22 (2.7)	
			827	
Marital status				
Single	276 (64.8)	299 (74.8)	575 (69.6)	**.002**
			826	
Work				
Unemployed	235 (55.0)	213 (53.5)	448 (54.3)	.662
			825	
Household size				
0–2 individuals	214 (51.7)	230 (59.3)	444 (55.4)	**.031**
>2individuals	200 (48.3)	158 (40.7)	358 (44.6)	
			802	
Income, Rands, median (IQR)	1295 (600–2970)	1900 (750–3000)	1200 (500–2000)	**<.001**
Binge drinking			698	
Yes	425 (97.0)	403 (97.3)	828 (97.8)	.784
			850	
Current smoker				
Yes	123 (28.7)	90 (22.5)	213 (25.7)	**.04**
			828	
Prison history				
Yes	21 (4.9)	42 (10.3)	63 (7.5)	**.003**
			837	
Miner (past or present)				
Yes	17 (4.0)	7 (1.7)	24 (2.9)	.053
			833	
Healthcare worker				
Yes	8 (1.9)	7 (1.7)	15 (1.8)	.889
			838	
TB contact				
Yes	54 (12.6)	51 (12.6)	105 (12.6)	.999
			836	
Previous TB				
Yes	196 (46.0)	129 (31.9)	325 (39.2)	**<.001**
			830	
Previous DM				
Yes	19 (4.4)	20 (4.9)	39 (4.7)	.717
			838	
HIV-1 status				
Uninfected	160 (36.5)	121 (29.4)	281 (33.1)	
Infected	242 (55.3)	277 (67.2)	519 (61.1)	
Unknown	36 (8.2)	14 (3.4)	50 (5.9)	
Total	438	412	850	
			850	
ART status, among those with HIV-1 infection				
Yes	166 (68.6)	89 (31.9)	255 (48.9)	**<.001**
			521	
Hypertension				
Yes	154 (35.2)	75 (18.1)	229 (26.9)	**<.001**
			850	
BMI				
<18 kg/m^2^ (underweight)	27 (6.6)	41 (10.3)	68 (8.4)	**<.001**
18–25 kg/m^2^ (normal)	224 (54.4)	277 (69.6)	501 (61.9)	
26–30 kg/m^2^ (overweight)	69 (16.8)	59 (14.8)	128 (15.8)	
>30 kg/m^2^ (obese)	92 (22.3)	21 (5.3)	113 (14.0)	
			810	
Waist circumference				
Raised (>94 cm males; >88 cm females)	144 (28.9)	53 (14.3)	167 (21.8)	**<.001**
			765	

Data are presented as no. (%) unless otherwise indicated. Number of participants is shown at the end of each category. Values in bold indicate statistical significance.

Abbreviations: ART, antiretroviral therapy; BMI, body mass index; HIV-1, human immunodeficiency virus type 1; IQR, interquartile range; TB, tuberculosis.

### Study Sample at Follow-up

Of the 850 patients enrolled, 639 returned for 3-month follow-up with 211 patients lost to follow-up (108 TB,103 non-TB) (Figure 1). Data comparing participants lost to follow-up to those followed up are presented in Supplementary Table 2.

### Glycemic Levels in TB Patients at Enrollment and Follow-up

Among TB patients with newly diagnosed DM, median HbA1c decreased at follow-up (5.7% vs 5.4%; *P* < .0001), although FPG in this group slightly increased (4.6 vs 4.7 mmol/L; *P* < .0064) at follow-up (Table 2). Among those with a preexisting diagnosis of DM, glycemic levels were sustained at high levels with no significant changes at follow-up (Table 2).

**Table 2.** Glycemic Levels Among Participants With Tuberculosis

	Enrollment (n = 412)			Follow-up (n = 304)		
Glycemic Marker	DM or IGR/Total	Prevalence (95% CI)	Median (IQR)	DM or IGR/Total	Prevalence (95% CI)	Median (IQR)
IGR						
HbA1c	147/363	40.7 (36.1–45.6)	6.0 (5.9–6.2)	39/276	12.8 (9.7–17.5)	5.9 (5.8–6.0)
FPG	40/363	10.9 (8.3–14.4)	5.8 (5.7–6.3)	29/276	10.5 (7.5–14.5)	5.3 (4.8–5.7)
HbA1c or FPG	177/363	48 (42.2–51.8)	…	62/276	21.8 (16.9–26.3)	…
Newly diagnosed DM and preexisting DM^a^						
HbA1c	42/410	10.2 (7.6–13.6)	7.6 (6.6–11.7)	16/296	5.4 (3.3–8.6)	10.3 (7.3–11.3)
FPG	18/410	4.4 (.3–6.9)	8.9 (7.8–12.0)	13/296	4.4 (2.6–7.5)	10.6 (7.2–15.4)
HbA1c or FPG	48/410	11.9 (9.10–15.4)	…	22/296	9.3 (6.4–13.0)	…
Newly diagnosed DM only						
HbA1c	25/390	6.4 (4.2–9.2)	6.7 (6.5–7)	6/284	2.1 (1.0–4.7)	6.6 (6.6–10.8)
FPG	9/390	2.3 (1.2–4.4)	8.7 (8.1–8.9)	4/284	1.4 (.5–3.7)	10.3 (7.1–14.5)
DM defined by HbA1c or FPG	28/390	7.1 (5.1–10.4)	…	9/284	2.9 (1.4–5.7)	…
Preexisting DM only						
HbA1c	18/20	89.5 (62.9–97.7)	11.2 (7.9–12.9)	10/14	71.4 (39.9–90.4)	9.3 (5.7–11.2)
FPG	9/20	45 (25.0–70.8)	6.9 (4.8–10.1)	9/14	64.3 (39.9–90.4)	8.7 (6.4–15.4)
HbA1c or FPG	20/20	100.00	…	14/14	100.00	…

Abbreviations: CI, confidence interval; DM, diabetes mellitus; FPG, fasting plasma glucose; HbA1c, glycated hemoglobin; IGR, impaired glucose regulation; IQR, interquartile range; TB, tuberculosis.

^a^Differences in denominator (n = 2 at enrollment and n = 8 at follow-up) due to exclusion of participants with missing DM diagnostic tests for either test.

### Prevalence of Diabetes and Impaired Glucose Regulation Among Patients With TB at Enrollment and Follow-up

The overall (including newly diagnosed DM and preexisting DM) prevalence of DM based on either HbA1c or FPG diagnostic tests was 11.9% (95% CI, 9.1%–15.4%) at enrollment and 9.3% (95% CI, 6.4%–13%) at follow-up (*P* = .273). The prevalence of IGR was 46.9% (95% CI, 42.15%–51.8%) and 21.5% (95% CI, 16.9%–26.3%) at follow-up (*P* < .001; Table 2). Among those with preexisting DM, glycemic levels were high (Table 2) beyond the cutoff for DM diagnosis at both enrollment and follow-up (Table 2).

### Glycemic Profile Among TB Patients

**Figure 2.**
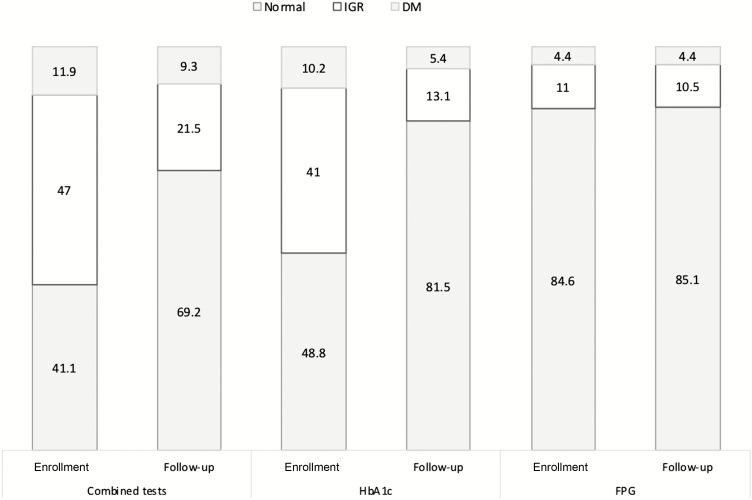
Changes in the prevalence (%) of hyperglycemia (IGR and DM) among patients with tuberculosis (TB) at enrollment (when TB diagnosed) and follow-up (3 months later). Abbreviations: DM, diabetes mellitus; FPG, fasting plasma glucose; HbA1c, glycated hemoglobin; IGR, impaired glucose regulation.

At enrollment, using both combined tests, 58.9% (n = 243) of TB patients were hyperglycemic (DM and IGR). This prevalence decreased to 30.8% (n = 94) at follow-up (Figure 2); 2.6% (n = 10) with DM at enrollment reverted to normal at follow-up; and 22.5% (n = 105) of patients with IGR reverted to normal at follow-up (Figure 2). With respect to diagnostic test, hyperglycemia detected by HbA1c was 51.2% (n = 186) enrollment and 18.5% (n = 65) at follow-up; and hyperglycemia detected by FPG was 15.4% (n = 38) at enrollment and 14.9% (n = 37) at follow-up.

### Association Between Hyperglycemia (DM and IGR) and TB at Enrollment and Follow-up

Irrespective of HIV-1 status, the overall TB/DM association was positive and significant at both enrollment (OR, 2.4 [95% CI, 1.3–4.3]) and at follow-up (OR, 3.3 [95% CI, 1.5–7.3]) but only when DM was defined by a positive result for either of the diagnostic tests or a previous DM history (Table 3). This significant association was not observed at either time point when DM diagnostic tests were used in isolation (Table 3).

**Table 3.** Association Between Tuberculosis and Diabetes Mellitus or Impaired Glucose Regulation at Enrollment and 3-Month Follow-up

	Enrollment			Follow-up		
Characteristic	Overall (N = 850)	Participants With HIV (n = 541)	Partcipants Without HIV (n = 309)	Overall (N = 639)	Participants With HIV (n = 399)	Participants Without HIV (n = 240)
IGR						
No IGR	1.0	1.0	1.0	1.0	1.0	1.0
IGR by HbA1c	**1.6 (1.1–2.3)**	1.5 (1.0–2.3)	**2.2 (1.2–4.1)**	0.6 (.4–1.1)	0.6 (.3–1.2)	0.8 (.3–2.0)
IGR by FPG	0.9 (.5–1.5)	1.2 (.6–2.2)	0.4 (.1–1.2)	1.2 (.6–2.3)	0.8 (.3–2.2)	1.6 (.6–4.2)
IGR by combined test (HbA1c or FPG)	**2.3 (1.6–3.3)**	**2.4 (1.51–3.8)**	**2.3 (1.1–4.7)**	0.84 (.53–1.36)	0.77 (.41–1.16)	1.10 (.47–2.57)
Combining preexisting DM and newly diagnosed DM						
No DM	1.0	1.0	1.0	1.0	1.0	1.0
DM by HbA1c	**2.4 (1.2–4.6)**	2.4 (1.0–5.9)	2.2 (.7–6.3)	2.1 (.8–5.3)	2.5 (.5–11.8)	1.8 (.5–6.7)
DM by FPG	2.3 (1.0–5.9)	2.9 (.7–12.2)	1.9 (.7–6.4)	2.8 (.9–8.4)	9.8 (.9–106.6)	1.6 (.4–6.5)
DM by combined test (HbA1c or FPG)	**2.8 (1.5–5.3)**	**2.4 (1.0–5.3)**	**3.5 (1.2–9.8)**	**3.3 (1.5–7.3)**	**3.8 (1.2–12.3)**	**3.5 (1.1–11.0)**
Newly diagnosed DM only						
No DM	1.0	1.0	1.0	1.0	1.0	1.0
DM by HbA1c	1.6 (.7–3.6)	1.7 (1.0–2.9)	1.0 (.1–7.6)	1.5 (.3–7.0)	1.3 (.2–8.1)	2.4 (.1–49.1)
DM by FPG	1.7 (.3–8.8)	9.2 (.9–97.0)	2.2 (1.0–4.7)	3.0 (.2–40.5)	^a^	2.0 (.6–7.2)
DM by combined test (HbA1c or FPG)	2.2 (1.0–4.7)	1.6 (.7–4.0)	3.6 (.7–19.5)	2.0 (.6–7.2)	1.6 (.3–7.3)	4.5 (.3–59.9)
Preexisting DM only						
No DM	1.0	1.0	1.0	1.0	1.0	1.0
DM	**3.7 (1.5–9.1)**	**6.3 (1.3–30.8)**	3.1 (.9–10.1)	**4.0 (1.6–10.1)**	**9.3 (1.7–49.0)**	3.0 (.9–10.1)

Data are presented as odds ratio (95% confidence interval). Bold text represents significant associations. Reference group for the associations: patients with no DM or IGR. All odds ratios were adjusted for sex, age, household size, income, hypertension (baseline), previous miner, previous prisoner, marital status, work status, and HIV-1 status.

Abbreviations: DM, diabetes mellitus; FPG, fasting plasma glucose; HbA1c, glycated hemoglobin; HIV, human immunodeficiency virus; IGR, impaired glucose regulation.

^a^Insufficient data to perform test-specific analysis.

The overall association between TB and IGR (by FPG or HbA1c) was positive at enrollment (OR, 2.3 [95% CI, 1.6–3.3]; Table 3) but not at follow-up (OR, 0.8 [95% CI, .5–1.4]). On further analysis by DM diagnostic test, the overall TB/IGR association at enrollment was significant when using the HbA1c test (OR, 1.6 [95% CI, 1.1–2.3]) but not by FPG (OR, 0.9 [95% CI, .5–1.5]) (Table 3).

### Comparison of the Association Between TB/Newly Diagnosed DM and TB/Preexisting DM

At enrollment, 4.5% (95% CI, 2.8%–6.8%; n = 20) of patients had preexisting DM. All these patients had poorly controlled DM as evidenced by high glycemic levels at enrollment and follow-up (Table 2). On restricting analysis to patients with newly diagnosed DM, the TB/DM association was significant at enrollment (OR, 2.12 [95% CI, 1.0–4.7]) but not at follow-up (Table 3). By contrast, the association between TB and preexisting DM was significant at both enrollment (OR, 3.7 [95% CI, 1.5–9.1]) and follow-up (OR, 4.0 [95% CI, 1.6–10.1]) (Table 3).

## DISCUSSION

We previously reported the prevalence of DM in patients with newly diagnosed TB and the cross-sectional TB/DM association at enrollment [12]. In this study, we investigated whether hyperglycemia identified in the patients with newly diagnosed TB at enrollment persisted after 3 months of TB treatment. We also assessed association between TB and hyperglycemia at both time points. This is the first study in South Africa, a setting of high TB and DM burden, to document transient hyperglycemia in TB patients with preexisting DM and newly diagnosed DM. Overall, we report significant association between TB and DM at both enrollment and follow-up and significant association between TB and IGR at enrollment but not follow-up. However, when these results were further analyzed by DM category (newly diagnosed vs preexisting) and by HIV status within these categories, differing patterns emerged. Given the socioeconomic conditions of our study setting, the patients with newly diagnosed DM in our study may represent the proportion of undiagnosed DM due to factors associated with limited access to DM screening and having health facilities overwhelmed by TB and HIV [17].

### Newly Diagnosed DM

Hyperglycemia, transient in the majority of participants with newly diagnosed DM and IGR, was predominantly accounted for by the latter at enrollment, and normalized at follow-up. Similar to our findings, other studies have shown frequent hyperglycemia in patients with TB at initiation of TB treatment, followed by normalization during treatment [23]. This may be due to inflammation in response to active TB [25–27], driven by complex interactions between hormones and cytokines [24].

Consistent with the literature, the association between DM/IGR and TB in newly diagnosed DM participants was only significant at enrollment but not at follow-up. With respect to the different diagnostic tests separately, none of the associations were significant.

### Preexisting DM

Patients with preexisting DM had poorly controlled disease at both diagnosis of TB and 3 months later, as reflected by both FPG and HbA1c at these timepoints. Unlike newly diagnosed DM participants, the significant TB/DM association at enrollment (OR, 3.7 [95% CI, 1.5–9.1]) in this group, which persisted at follow-up (OR, 4.0 [95% CI, 1.6–10.1]), reflects poor glycemic control between the study timepoints. Hyperglycemia may have been exacerbated by acute TB. The persistence of raised glycemic levels reflects inadequate management of these patients, possibly due to poor follow-up, and highlights the complexity of clinical care in this subgroup.

A Korean retrospective study showed that TB is usually diagnosed 1 year following the diagnosis of DM [27]. Therefore, the observed relative odds of preexisting DM among patients with TB compared to those without TB in this study (OR, 2.8 [95% CI, 1.5–5.3]) may approximate the relative risk to develop TB among patients with diabetes. Although the temporal relationship between TB and DM remains contentious, irrespective of the causal direction, comorbidity of TB and DM increases the risk of adverse TB treatment outcomes including treatment failure, mortality, and drug resistance [3, 5, 13]. The observed poorly managed DM in these individuals with TB also highlights the increased risk of adverse TB treatment outcomes among these patients.

### TB, DM, and HIV

When stratified by HIV-1 status, the TB/DM association (when DM is defined by the different diagnostic tests separately) was not significant at either time point. Interaction between HIV, TB and DM is still not well understood and different studies report varying results.

It is complicated to interpret the interplay between TB, HIV, and DM as a wide range of factors may influence what is observed. This includes the effect of ART, HIV-1 infection as an independent risk factor for both DM and TB, or the choice of diagnostic test. Cotrimoxazole, administered to people living with HIV-1, can lead to hypoglycemia [28]. Conversely, ART, particularly regimens containing protease inhibitors, increases insulin resistance, thus increasing the risk of diabetes [29].

Other studies suggest that HbA1c may underestimate the presence of hyperglycemia in people living with HIV-1 and that this may be due to nucleoside reverse transcriptase inhibitor use [30, 31]. In our study, HbA1c detected a higher prevalence of hyperglycemia. In a 2015 review by English et al, it was suggested that an HbA1c test is likely to be affected by iron deficiency anemia and may result in spurious increases of HbA1c level [32]. On the other hand, non–iron deficiency anemia may lead to a reduction in HbA1c levels [32, 33]. A recent study observed lower HbA1c mean levels in severely anemic patients; however, due to a limited sample, no further analysis was performed to explore this relationship [34]. The effect of anemia on the direction of the association is therefore unclear.

We explored the potential effect of unmeasured confounding on our results by performing sensitivity analysis (Supplementary Table 3) [35]. This showed that in IGR and newly diagnosed DM, the association with TB was rendered nonsignificant at baseline and follow-up. As such we note that weaker residual confounding may explain away our observed estimates. However, the association between TB and preexisting DM was significant both at enrollment and follow-up with ORs ranging between 3.7 and 9.3. To explain away these associations, an unmeasured confounder would need to be associated with both TB and preexisting DM with risk ratios ranging between 2.9 and 6.9, but weaker confounding would not.

There were strengths and limitations to this study. A limited number of studies have evaluated hyperglycemia in individuals with TB, particularly in Africa where there is a high prevalence of comorbidity with other diseases such as HIV-1. TB cases were diagnosed according to South African guidelines [19], with the Gene Xpert analyzed in a centralized national health laboratory. For enrollment and follow-up, DM measurements were performed using 2 recommended tests. We relied on medical records to document ART use, as such we could not reliably ascertain duration of ART use in our multivariate analysis.

Our study follow-up time was limited to 3 months and we were not able to analyze the effect of hyperglycemia on TB outcomes. Because critically ill patients were excluded in the study, it may have biased the study population to appear healthier.

The proportion of participants lost to follow-up in this study, and to the health system, was relatively high (n = 212 [24.9%]) (Supplementary Table 2). Those followed up were older, mostly unemployed, and likely to have known DM and hypertension. Our results at follow-up may thus be slightly biased as they represent an older population prone to chronic conditions. Reasons for loss to follow-up include migration to other parts of the country and transfer to other health facilities in the province. To reduce bias and loss of statistical power, we imputed HIV status for patients with unknown HIV status. Therefore, loss to follow-up is less likely to have biased the associations observed in the study. The loss to follow-up observed in this study reflects how patients are lost in healthcare systems in this setting. This therefore highlights the importance of improving retention of patients in care for optimal management of all chronic diseases.

## CONCLUSIONS

This is the first study in the South African context of high TB/HIV and rapidly increasing DM burdens to describe changes in glucose levels among patients with TB during treatment. This study showed that hyperglycemia was common in TB patients with DM. This confirms the need for confirmation DM tests in TB patients during and/or after the course of TB treatment. The association between DM and TB persisted at follow-up in participants with preexisting DM, particularly those infected with HIV-1. This highlights an important need for improved co-management of TB, DM, and HIV to limit the risks of adverse outcomes.

## Supplementary Data

Supplementary materials are available at *Clinical Infectious Diseases* online. Consisting of data provided by the authors to benefit the reader, the posted materials are not copyedited and are the sole responsibility of the authors, so questions or comments should be addressed to the corresponding author.

ciz928_suppl_Supplementary_Tables

## Notes

***Author contributions.*** T. O. designed the study and was principal investigator. T. O., N. S. L., and R. J. W. were involved in the design of the study. M. K. led the manuscript draft, data analysis, and data interpretation. All authors were involved in draft and critical review of the manuscript. N. B. led participant recruitment, retention, phenotypic characterization of all participants, and data management. R. G. led study coordination and contributed to patient recruitment, follow-up, and collection of data. The final manuscript draft was approved by all authors. T. O. had full access to all of the data in the study and takes responsibility for the integrity of the data and the accuracy of the data analysis.

***Disclaimer.*** The funders played no role in the design, conduct, or analysis of the study.

***Financial support.*** This work was supported by the Wellcome Trust (grant numbers 084323, 104873, and 203135), a Carnegie Corporation Postdoctoral Fellowship, and a Harry Crossley Senior Clinical Fellowship. R. J. W. is supported by the Francis Crick Institute, which receives funding from Cancer Research UK (grant number FC001010218), Research Councils UK (grant number FC0010218), and the Wellcome Trust (grant number FC0010218). He also receives support from the National Institutes of Health (NIH) (grant number U1 AI115940), NIH (grant number WILK116PTB), and European and Developing Countries Clinical Trials Partnership (grant number SRIA 2015–1065). M. K. is supported by the South African Centre for Epidemiological Modelling and Analysis, the International Epidemiology Databases to Evaluate AIDS, and the NIH (grant number U01AI069924).

***Potential conflicts of interest.*** The authors report no potential conflicts of interest. All authors have submitted the ICMJE Form for Disclosure of Potential Conflicts of Interest. Conflicts that the editors consider relevant to the content of the manuscript have been disclosed.
