# Supplementary material for: Tuberculosis, Human Immunodeficiency Virus, and the Association With Transient Hyperglycemia in Periurban South Africa
Source: Clin Infect Dis. 2019 Sep 26;71(4):1080–8. doi: 10.1093/cid/ciz928 (PMC7428387; doi:10.1093/cid/ciz928)
Supplement: ciz928_suppl_Supplementary_Tables [file ciz928_suppl_supplementary_tables.docx]

**Supplementary Table 1:** comparing complete case and imputed analyses for multi-variate analysis results on the association between TB and IGR/DM.

Imputation using the chained equations method was implemented in STATA using the *ice* command. Five imputed datasets were created. The *mi* STATA command was used to perform logistic regression analysis on the combined imputed datasets. The base model included *a priori* confounding variables of age and sex. Variables associated with TB (p<0.10) on univariable analysis were then added, sequentially including variables that improved the model based on significant lowering of the Akaike Information Criterion (AIC) in non-nested model comparisons. Potential effect modification between variables was determined by exploring the statistical significance of interaction variables. The fit of the model was assessed using Pearson’s goodness-of-fit.

|  | Imputed data | | | Original data | |
| --- | --- | --- | --- | --- | --- |
|  | Overall (N= 850) | | | Overall (N = 802) | |
|  | HIV (n= 543) | | | HIV (n = 521) | |
|  | HIV uninfected (n= 309) | | | HIV uninfected (n = 281) | |
|  |  | Enrolment | Follow-up | Enrolment | Follow-up |
|  |  | Odds Ratio (95% CI) | Odds Ratio (95% CI) | Odds Ratio (95% CI) | Odds Ratio (95% CI) |
| TB and IGR association | | | | | |
| HbA1c | Overall | 1.6 (1.1 - 2.3) | 0.6 (0.4 - 1.1) | 1.6 (1.1 – 2.2) | 0.6 (0.4 – 1.1) |
|  | HIV | 1.5 (1.0 - 2.3) | 0.6 (0.3 -1.2) | 1.5 (1.0 – 2.5) | 0.6 (0.3 – 1.2) |
|  | HIV-uninfected | 2.2 (1.2 - 4.1) | 0.8 (0.3 – 2.0) | 2.4 (1.2 – 4.6) | 0.8 (0.3 – 2.0) |
| FPG | Overall | 0.9 (0.5 - 1.5) | 1.2 (0.6- 2.3) | 0.9 (0.5 – 1.5) | 1.1 (0.6 -2.2) |
|  | HIV | 1.2 (0.6 - 2.2) | 0.8 (0.3 - 2.2) | 1.0 (0.5 – 2.0) | 0.8 (0.3 – 2.4) |
|  | HIV-uninfected | 0.4 (0.1 - 1.2) | 1.6 (0.6 - 4.2) | 0.3 (0.8 - 1.0) | 1.3 (.05 – 3.5) |
| HbA1c or FPG | Overall | 2.3 (1.6 - 3.3) | 0.8 (0.5 - 1.4) | 2.3 (1.5 – 3.4) | 0.8 (0.5 – 1.3) |
|  | HIV | 2.4 (1.51 - 3.8) | 0.8 (0.4 - 1.2) | 2.2 (1.4 -3.6) | 0.8 (0.4 – 1.5) |
|  | HIV-uninfected | 2.3 (1.1 - 4.7) | 1.1 (0.5 - 2.6) | 2.2 (1.1 – 4.3) | 1.0 (0.4 – 2.4) |
| TB and Pre-existing DM and newly diagnosed DM combined | | | | | |
| HbA1c | Overall | 2.4 (1.2 - 4.6) | 2.1 (0.8 - 5.3) | 2.4 (1.2 - 4.6) | 2.0 (0.8 – 5.1) |
|  | HIV | 2.4 (1.0 - 5.9) | 2.5 (0.5 - 11.8) | 1.8 (0.7 – 4.6) | 2.2 (0.46 – 11.0) |
|  | HIV-uninfected | 2.2 (0.7 - 6.3) | 1.8 (0.5 - 6.7) | 1.5 (0.5 – 4.7) | 1.7 (4.6 – 6.3) |
| FPG | Overall | 2.3 (1.0 - 5.9) | 2.8 (0.9 - 8.4) | 2.2 (0.9 – 5.4) | 2.6 (0.7 – 8.0) |
|  | HIV | 2.9 (0.7 - 12.2) | 9.8 (0.9 - 106.6) | 3.4 (0.7 – 17.8) | * |
|  | HIV-uninfected | 1.9 (0.7 - 6.4) | 1.6 (0.4- 6.5) | 2.0 (0.6 – 6.9) | 1.5 0.4 – 6.2) |
| Combined (HbA1c or FPG) | Overall | 2.8 (1.5 - 5.3) | 3.3 (1.5 - 7.3) | 2.6 (0.8 - 4.5) | 3.2 (1.5 – 7.2) |
|  | HIV | 2.4 (1.0 - 5.3) | 3.8 (1.2 - 12.3) | 1.9 (0.8 – 4.6) | 3.8 (1.0 – 14.0) |
|  | HIV-uninfected | 3.5 (1.2 - 9.8) | 3.5 (1.1 - 11.0) | 2.6 (0.9 – 7.7) | 3.0 (0.9 – 9.7) |
| TB and Newly diagnosed DM (only) association | | | | | |
| HbA1c | Overall | 1.6 (0.7 - 3.6) | 1.5 (0.3 - 7.0) | 1.7 (0.7 – 3.7) | 1.6 (0.4 – 7.7) |
|  | HIV | 1.7 (1.0 2.9) | 1.3 (0.2 - 8.1) | 1.3 (.5 – 3.6) | 1.1 (0.2 – 7.3) |
|  | HIV-uninfected | 1.0 (0.1 - 7.6) | 2.4 (.1 - 49.1) | 0.51 (0.04 – 6.2) | 2.01 (0.1 – 40.0) |
| FPG | Overall | 3.1 (0.8 - 12.3) | 4.6 (0.4 -51.6) | 3.2 (0.9 – 12.3) | 4.8 (0.41 – 55.1) |
|  | HIV | 1.7 (0.3 - 8.8) | 3.0 (0.2 - 40.5) | 1.7 (0.27 – 11.8) | * |
|  | HIV-uninfected | 9.2 (0.9 - 97.0) | * | 8.5 (0.9 – 84.7) | * |
| Combined (HbA1c or FPG) | Overall | 2.2 (1.0 - 4.7) | 2.0 (0.6 - 7.2) | 2.1 (1.0 – 4.6) | 2.0 (0.57 – 7.3) |
|  | HIV | 1.6 (0.7 - 4.0) | 1.6 (0.3 - 7.3) | 1.3 (0.5 – 3.6) | 1.8 (0.3 -10.5) |
|  | HIV-uninfected | 3.6 (0.7 - 19.5) | 4.5 (0.3 - 59.9) | 2.7 (0.4 – 16.0) | 3.8 (0.3 – 48.6) |
| TB/pre-existing DM association. Insufficient data to stratify by test | | | | | |
| Combined (HbA1c or FPG) | Overall | 3.7 (1.5 - 9.1) | 4.0 (1.6 - 10.1) | * | * |
|  | HIV | 6.3 (1.3 - 30.8) | 9.3 (1.7 - 49.0) | * | * |
|  | HIV-uninfected | 3.1 (0.9 - 10.1) | 3.0 (0.9 - 10.1) | * | * |

IGR: impaired glucose regulation; HbA1c Glycated haemoglobin. FPG: fasting plasma glucose. DM: diabetes mellitus. OR: odds ratios. CI: confidence interval. All odds ratios were adjusted for sex, age, household size, income, hypertension (baseline), previous miner, previous prisoner, marital status, work status and HIV-1 status. Reference group for the associations: patients with no DM or IGR. *: insufficient data.

**Supplementary Table 2:** Participant characteristics by follow-up and loss to follow up.

|  | **Follow up**  **(N = 639)** | **Lost to follow up (N = 211)** | **Totals**  **(N = 850)** | **P-value** |
| --- | --- | --- | --- | --- |
| TB cases | 303 (47.49) | 109 (51.42) | 412 (48.47) | 0.322 |
| Non TB Cases | 335 (52.51) | 103 (48.58) | 438 (51.53) |  |
| No DM | 567 (88.87) | 196 (92.45) | 763 (89.76 ) | 0.136 |
| DM | 71 (11.13) | 16 (7.55) | 87 (10.24) |  |
| FBG (median; IQR) | 4.7 (5.2 - 4.3) | 4.7 (5.2 - 4.4) | 4.7 (4.3 - 5.20 | 0.5111 |
| HbA1c (median; IQR) | 5.7 (5.5 -6) | 5.7 (5.5 - 6) | 5.7 (5.5 - 6) | 0.7417 |
| Age |  |  |  |  |
| 0-24 | 39 (6.11) | 20 (9.43) | 59 (6.94 ) | 0.001 |
| 25-34 | 190 (29.78) | 87 (41.04) | 277 (32.59 ) |  |
| 35-44 | 201 (31.50) | 61 (28.77) | 262 (30.82 ) |  |
| 45-54 | 123 (19.28) | 25(11.79) | 148 (17.41) |  |
| >55 | 85 (13.32) | 19 (8.96) | 104 (12.24 ) |  |
| Age (median; IQR) | **39 (31.4 - 48.1)** | **34.9 (29.4 - 43)** | **38.1 (30.9 - 47.1)** | **0.0001** |
| Female | 301 (47.48) | 96 (45.28) | 397 (46.93 ) | 0.580 |
| Education level |  |  |  |  |
| up to primary | 196 (31.26) | 62 (31.16) | 258 (31.23 ) | 0.690 |
| up to secondary | 416 (66.35) | 130 (65.33) | 546 (66.10) |  |
| higher education | 15 (2.39) | 7 (3.52) | 22 (2.66) |  |
| Marital status (single) | 425 (68.00) | 149 (74.50) | 574 (69.58) | 0.082 |
| Unemployed | **353 (56.57)** | **106 (47.00)** | **447 (54.25)** | **0.018** |
| Household size |  |  |  |  |
| 0–2 individuals | 331 (54.53) | 112 (57.73) | 443 (55.31 ) | 0.435 |
| >2 individuals | 276 (45.47) | 82 (42.27) | 358 (44.69 |  |
| Income categories ZAR n=730 |  |  |  |  |
| no income | 21 (3.83) | 9 (5.00) | 30 (4.12 ) | 0.799 |
| R 1 – R 1 600 | 313 (57.01) | 94 (52.22) | 407 (55.83) |  |
| R 1 601 – R 3 200 | 128 (23.32) | 46 (25.56) | 174 (23.87 ) |  |
| R 3 201 – R 6 400 | 71 (12.93) | 25 (13.89) | 96 (13.17 ) |  |
| R 6 401 – R 12 800 | 14 (2.55) | 6 (3.33) | 20 (2.74 ) |  |
| R 12 801 or more | 2 (0.36) | 0 (0.00) | 2 (0.27) |  |
| R 6 401 – R 12 800 | 14 (2.55) | 6 (3.33) | 20 (2.74 ) |  |
| R 12 801 or more | 2 (0.36) | 0 (0.00) | 2 (0.27) |  |
| Binger | 617 (96.71) | 209 (98.58) | 826 (97.18) | 0.153 |
| Smoker | 161 (25.72) | 52 (25.87) | 213 (25.76) | 0.966 |
| Prison history | 46 (7.34) | 17 (8.13) | 63(7.54 ) | 0.705 |
| Miner | 18 (2.89) | 6 (2.87) | 24 (2.88 ) | 0.989 |
| Health care worker | 13 (2.07 | 2 (0.96) | 15 (1.79 ) | 0.293 |
| TB contact | 79 (12.62) | 26 (12.44) | 105 (12.57 ) | 0.946 |
| Previous TB | 250 (40.00) | 74 (35.92) | 324 (38.99 ) | 0.427 |
| Previous diabetes | **39 (6.21)** | **0 (0.0)** | **39 (4.66)** | **0.00** |
| HIV-1 status |  |  |  |  |
| Uninfected | 223 (35.62) | 61 (29.76) | 284 (34.18 ) | 0.275 |
| Infected | 361 (57.67) | 127 (61.95) | 488 (58.72) |  |
| Unknown | 42 (6.71) | 17 (8.29) | 59 (7.10 ) |  |
| ART |  |  |  |  |
| Hypertension | **187 (29.31)** | **42 (19.81)** | **229 (26.94)** | **0.007** |
| Raised waist circumference | 129 (22.43) | 37 (19.58) | 166 (21.73 ) | 0.408 |
| waist circumference (median; IQR) | **82 (91 - 75)** | **79 (88 - 75)** | **81 (91 - 75)** | **0.0655** |
| BMI categories |  |  |  |  |
| 18-25 | 377 (67.08) | 124 (69.27) | 501 (67.61 ) | 0.113 |
| 26-30 | 92 (16.37) | 36 (20.11) | 128 (17.27 ) |  |
| >30 | 93 (16.55) | 19 (10.61) | 112 (15.11 ) |  |
| BMI (media; IQR) | 22.72 (20 - 27.28) | 22.26 (19.86) | 22.57 (27.07 -19.86) | 0.1601 |

**Supplementary Table 3:** Sensitivity analysis for the observed TB and DM/OR association using e-values for odds ratios from our analysis, converted to risk ratios.

|  |  | E-value for point estimated | | | | | | E-value for lower CI | | | | | |
| --- | --- | --- | --- | --- | --- | --- | --- | --- | --- | --- | --- | --- | --- |
|  |  | Enrolment | Follow - up | Enrolment | Follow - up | Enrolment | Follow - up | Enrolment | Follow - up | Enrolment | Follow - up | Enrolment | Follow - up |
|  |  | OR | OR | Converted OR to RR | Converted OR to RR | E-value for the estimate | E-value for the estimate | OR | OR | Converted OR to RR | Converted OR to RR | E-value for the estimate | E-value for the estimate |
| TB and DM association. No DM is the refence group. | | | | | | | | | | | | | |
| HbA1c | Overall | 2,4 | 2,1 | 1,5 | 1,2 | 2,3 | 4 | 1,2 | 0,8 | 1,1 | 0,9 | 1,4 | 1,0 |
|  | HIV | 2,4 | 2,5 | 1,5 | 1,2 | 2,3 | 4 | 1 | 0,5 | 1,0 | 0,7 | 1,0 | 1,0 |
|  | HIV-uninfected | 2,2 | 1,8 | 1,5 | 1,2 | 2,2 | 3,8 | 0,7 | 0,5 | 0,8 | 0,7 | 1,0 | 1,0 |
| FPG | Overall | 2,3 | 2,8 | 1,5 | 1,2 | 2,2 | 3,9 | 1 | 0,9 | 1,0 | 0,9 | 1,0 | 1,0 |
|  | HIV | 2,9 | 9,8 | 1,7 | 1,3 | 2,5 | 4,5 | 0,7 | 0,9 | 0,8 | 0,9 | 1,0 | 1,0 |
|  | HIV-uninfected | 1,9 | 1,6 | 1,4 | 1,2 | 2 | 3,4 | 0,7 | 0,4 | 0,8 | 0,6 | 1,0 | 1,0 |
| Combined (HbA1c or FPG) | Overall | 2,8 | 3,3 | 1,7 | 1,3 | 2,5 | 4,4 | 1,5 | 1,5 | 1,2 | 1,2 | 1,7 | 1,7 |
|  | HIV | 2,4 | 3,8 | 1,5 | 1,2 | 2,3 | 4 | 1 | 1,2 | 1,0 | 1,1 | 1,0 | 1,4 |
|  | HIV-uninfected | 3,5 | 3,5 | 1,9 | 1,4 | 2,8 | 5,1 | 1,2 | 1,1 | 1,1 | 1,0 | 1,4 | 1,3 |
| The association between TB and IGR association. No IGR is the refence group. | | | | | | | | | | | | | |
| HbA1c | Overall | **1,6** | 0,6 | 1,3 | 1,1 | 1,8 | 3 | 1,1 | 0,4 | 1,0 | 0,6 | 1,3 | 1,0 |
|  | HIV | 1,5 | 0,6 | 1,2 | 1,1 | 1,7 | 2,8 | 1 | 0,3 | 1,0 | 0,5 | 1,0 | 1,0 |
|  | HIV-uninfected | 2,2 | 0,8 | 1,4 | 1,2 | 2,1 | 3,5 | 1,2 | 0,3 | 1,1 | 0,5 | 1,4 | 1,0 |
| FPG | Overall | 0,9 | 1,2 | 0,9 | 1 |  | 1 | 0,5 | 0,6 | 0,7 | 0,8 | 1,0 | 1,0 |
|  | HIV | 1,2 | 0,8 | 1,1 | 1 | 1,4 | 2,2 | 0,6 | 0,3 | 0,8 | 0,5 | 1,0 | 1,0 |
|  | HIV-uninfected | 0,04 | 1,6 | 0,2 | 0,4 |  |  | 0,1 | 0,6 | 0,3 | 0,8 | 1,0 | 1,0 |
| Combined (HbA1c or FPG) | Overall | 2,3 | 2,3 | 1,5 | 1,2 | 2,2 | 3,9 | 1,6 | 1,6 | 1,3 | 1,3 | 1,8 | 1,8 |
|  | HIV | 2,4 | 0 | 1,5 | 1,2 | 2,3 | 4 | 1,5 | 1,5 | 1,2 | 1,2 | 1,7 | 1,7 |
|  | HIV-uninfected | 2,3 | 1,1 | 1,5 | 1,2 | 2,2 | 3,9 | 1,1 | 1,5 | 1,0 | 1,2 | 1,3 | 1,7 |
| The association between TB and newly diagnosed DM association. No IGR is the refence group. | | | | | | | | | | | | | |
| HbA1c | Overall | 1,6 | 1,5 | 1,3 | 1,1 | 1,8 | 3 | 0,7 | 0,3 | 0,8 | 0,5 | 1,0 | 1,0 |
|  | HIV | 1,7 | 1,3 | 1,3 | 1,1 | 1,9 | 3,1 | 1 | 0,2 | 1,0 | 0,4 | 1,0 | 1,0 |
|  | HIV-uninfected | 1 | 2,4 | 1 | 1 | 1 | 1 | 0,1 | 0,1 | 0,3 | 0,3 | 1,0 | 1,0 |
| FPG | Overall | 3,1 | 4,6 | 1,8 | 1,3 | 2,6 | 4,7 | 0,3 | 0,2 | 0,5 | 0,4 | 1,0 | 1,0 |
|  | HIV | 1,7 | 3 | 1,3 | 1,1 | 1,9 | 3,1 | 0,9 | * | 0,9 |  | 1,0 | 1,0 |
|  | HIV-uninfected | 9,2 | * | 3 | * | 4,5 | * | 1 | 0,6 | 1,0 | 0,8 | 1,0 | 1,0 |
| Combined (HbA1c or FPG) | Overall | 2,2 | 2 | 1,5 | 1,2 | 2,2 | 3,8 | 1 | 0,6 | 1,0 | 0,8 | 1,0 | 1,0 |
|  | HIV | 1,6 | 1,6 | 1,3 | 1,1 | 1,8 | 3 | 0,7 | 0,3 | 0,8 | 0,5 | 1,0 | 1,0 |
|  | HIV-uninfected | 3,6 | 4,5 | 1,9 | 1,4 | 2,8 | 5,1 | 0,7 | 0,3 | 0,8 | 0,5 | 1,0 | 1,0 |
| The association between TB and pre-existing DM association. Not enough data to stratify by test. No IGR is the refence group | | | | | | | | | | | | | |
| Combined (HbA1c or FPG) | Overall | 3,7 | 4 | 1,9 | 1,4 | 2,9 | 5,2 | 1,5 | 1,6 | 1,2 | 1,3 | 1,7 | 1,8 |
|  | HIV | 6,3 | 9,3 | 2,5 | 1,6 | 3,7 | 6,9 | 1,3 | 1,7 | 1,1 | 1,3 | 1,5 | 1,9 |
|  | HIV-uninfected | 3,1 | 3 | 1,8 | 1,3 | 2,6 | 4,7 | 0,9 | 0,9 | 0,9 | 0,9 | 1,0 | 1,0 |

IGR: impaired glucose regulation; HbA1c Glycated haemoglobin. FPG: fasting plasma glucose. DM: diabetes mellitus. CI: confidence interval. OR: odds ratios. RR: risk ratio. All odds ratios were adjusted for sex, age, household size, income, hypertension (baseline), previous miner, previous prisoner, marital status, work status and HIV-1 status. *: insufficient data. Shaded cells are the associations which were significant in the multivariate analysis.

Conversion of odds ratio (OR) to risk ratio (RR): $RR\approx\sqrt{OR}$ . E-value $=RR+\sqrt{RR(RR-1)}$

*The aim of our study was to assess transient hyperglycaemia in TB patients. For pre-existing DM, we observed that associations were persistently strong both at enrolment and follow-up. We observed that hyperglycaemia induced by TB was indicated by the higher prevalence of IGR and newly diagnosed DM at enrolment which reduced at follow-up; and the stronger IGR- and newly-diagnosed-DM- TB associations at enrolment, which dissipated at follow-up. We assert that normalisation of the hyperglycaemic state was due to TB treatment over the three months period. Nonetheless, there could have been some unmeasured confounders that may have influenced our results such as the use of other hypoglycaemic agents, and the effect of severe anaemia on HbA1c.*

*To explore the potential effect of unmeasured confounding on our results, particularly what could have led to reductions in glycaemic levels, we performed sensitivity analysis by calculating e-values for the associations we observed. This e-value measure represents the minimum strength of association that an unmeasured confounder would need to have with both TB (our outcome), and DM/IGR (our exposure variable), conditional on the other measured covariates, to fully explain away the DM-TB and IGR-TB associations we observed in our study (7). Based on suggestions that diagnostic tools, particularly HbA1c may be influenced by anaemia, anaemia – iron deficient or non-iron deficient – may be a potential confounder. However, we cannot ascertain the direction of influence on the measured associations.*

*Associations between IGR and TB across HIV strata:*

- - - - *IGR by HbA1c in HIV-uninfected patients; and IGR defined by both HbA1c and FPG, were only significantly associated with TB at enrolment. For the associations at enrolment, we observed e-values ranging between 1.4 and 3.9 on the risk ratio scale. To explain away the associations we observed, an unmeasured confounder would need to be associated with both TB and IGR with risk ratios of between 1.4 and 3.9.*
      - *Due to lack of significance of these results, particularly at follow-up, we note that these associations are not robust, and that weaker confounding may explain away our observed estimates.*
      - *Although there are studies which suggest that HbA1c is lower in iron deficient patients, there are limited epidemiological studies that show the magnitude of the association between TB and/or DM in the context of iron-deficiency.*

*Association between newly diagnosed DM and TB, across HIV strata:*

*None of the associations were significantly different from 1.0. Although the magnitude of the associations are in the positive direction, we can make no assertions from these results due to lack of significance.*

*Association between pre-existing DM and TB, across HIV strata:*

*There was insufficient data to perform diagnostic test specific analysis. The associations were significant only in HIV-infected patients both at enrolment at and follow up. The e-values ranged between 2.9 and 6.9 on the risk ratio scale. To explain away these observed associations, an unmeasured confounder would need to be associated with TB and DM with risk ratios between 2.9 and 6.9, but weaker confounding would not.*
